# The influence of overweight and obesity in the oral health of a sample of portuguese adults

**DOI:** 10.1093/eurpub/ckac131.555

**Published:** 2022-10-25

**Authors:** N Veiga, L Ferreira, P Couto, P Correia, P Lopes, MJ Correia, I Coelho

**Affiliations:** 1 Centre Interdisciplinary Research in Health, Universidade Católica Portuguesa, Viseu, Portugal; 2 Faculty of Dental Medicine, Universidade Católica Portuguesa, Viseu, Portugal; 3 Family Health Unit Grão Vasco, Health Centre III, Viseu, Portugal

## Abstract

**Background:**

Overweight and obesity may lead to different problems in various body systems and in the oral cavity. Dental caries and periodontal disease have been related with overweight. The aim of this study was to assess how overweight and obesity have impact on the lifestyle, oral habits and oral pathologies.

**Methods:**

We conducted an observational cross-sectional study where we applied a questionnaire to 140 individuals from Lisbon and Viseu, Portugal., which 70 had a normal Body Mass Index (BMI) (control group) and 70 had an excessive BMI. We also made an oral observation in each individual to record the permanent teeth decayed, missing and filled index (DMFT), the Community Periodontal Index and the oral hygiene status.

**Results:**

From the final sample, 30% of the subjects with overweight brushed their teeth once a day or less, while the majority (62.9%) of the control group brushed twice a day. In the oral examination, 70% had calculus, while in the control group only 22.5% presented calcified plaque. The DMFT was higher among the obesity group in comparison with the control group. Regarding periodontal disease, the participants with overweight need more dental intervention (81.4%) in contrast with the control group (14%).

**Conclusions:**

Most overweight and obese individuals present precarious oral hygiene habits, higher prevalence of dental caries, and worse periods of periodontal health. They are not aware of the repercussions of the association between their cariogenic diet, oral health and overweight.

**Key messages:**

• Oral health behaviors are related with other health conditions, namely obesity and this is a fundamental public health issue.

• Primary preventive strategies should be established having in consideration the oral health status of adults in treatment for obesity and in weight control programs.

